# βC1 of chili leaf curl betasatellite is a pathogenicity determinant

**DOI:** 10.1186/1743-422X-8-509

**Published:** 2011-11-08

**Authors:** Muhammad N Tahir, Shahid Mansoor

**Affiliations:** 1 Agricultural Biotechnology Division, National Institute for Biotechnology and Genetic Engineering, Faisalabad, Pakistan

**Keywords:** Begomoviruses, betasatellite, Cotton leaf curl Multan betasatellite, Chili leaf curl betasatellite

## Abstract

**Background**

Cotton leaf curl disease in the Indian subcontinent is associated with several distinct begomoviruses that interact with a disease-specific DNA satellite named Cotton leaf curl Multan betasatellite (CLCuMB). However, we have recently reported that Chili leaf curl betasatellite (ChLCB) is also occasionally found associated with the disease in Pakistan. The question as to whether ChLCB contributes to the development of disease symptoms such as leaf curling and enations remain to be answered. We have previously shown that the expression of βC1 of CLCuMB develops all symptoms of cotton leaf curl disease in *Nicotiana benthamiana *when expressed from PVX vector.

**Findings**

The role of ChLCB in the induction of typical disease symptoms was studied by its expression from PVX vector in *N. benthamiana*. The expression of βC1 from PVX vector developed severe leaf curl symptoms and leaf-like enations that resemble the phenotype induced by βC1 of CLCuMB.

**Conclusions**

The results presented here show that the expression of βC1 of ChLCB from PVX vector exhibit phenotype typical of cotton leaf curl and therefore ChLCB may contribute to the disease symptoms.

## Findings

Cotton leaf curl disease is the most important constraint on cotton production in Pakistan and northern India. The disease is transmitted by whitefly and is caused by a begomovirus disease complex [1]. Begomoviruses, transmitted by the vector *Bemisia tabaci*, are the most destructive single-stranded plant DNA viruses (family *Geminiviradae*) that cause huge yield losses to many dicotyledonous crops worldwide. The genome of begomoviruses comprises of either two components (bipartite) recognized as DNA-A and DNA-B, each of which is approximately 2.8 kb or a single component (monopartite) which is homologous to DNA-A of bipartite begomoviruses. Except for some tomato-infecting begomoviruses, the majority of monopartite begomoviruses are associated with DNA satellite molecules known as betasatellites [2]. The vast majority of begomovirus-betasatellite complexes are also associated with another DNA satellite named as alphasatellite (formerly known as DNA 1). Although alphasatellites are not essentially required to cause disease symptoms, their ubiquitous presence throughout the Old World and recently in the New World is intriguing. Recent results suggest that they may contribute to disease induction by overcoming host defense mediated by RNA silencing [3].

Betasatellites are single-stranded, symptom modulating, pathogenicity determinant DNA satellites [4]. They depend on their helper viruses for replication, encapsidation and transmission by the insect vector and are often required by their helper viruses for symptom induction in their original hosts [2,5,6]. Disease complexes that consist of monopartite begomoviruses and betasatellite form the largest group of begomoviruses. Sequence analyses of betasatellites showed that they consist of a single open reading frame (βC1), an adenine rich region and a highly conserved region known as satellite conserved region (SCR) [7]. βC1, a 13.5 kDa protein, which is encoded in complementary-sense orientation, is a pathogenicity determinant [4,8-10], a suppressor of RNA silencing [11-13] and can also replace the movement function of DNA-B of a bipartite begomovirus [14].

Betasatellites show host specificity and can be divided into two major groups; one infecting malvaceous hosts and the other infecting non-malvaceous hosts [7]. For example a single species of betasatellite named as Cotton leaf curl Multan betasatellite (CLCuMB) is associated with cotton leaf curl disease in Pakistan and India [5]. Similarly, a single species of betasatellite is associated with leaf curl disease of cotton and okra in Africa. The association of a distinct betasatellite with chili leaf curl disease in Pakistan has been reported previously [7]. The diversity analysis of the betasatellites from symptomatic chili pepper from Punjab and Khyber Pakhtoonkhwa provinces of Pakistan revealed that a single species of betasatellite named as Chili leaf curl betasatellite (ChLCB) is prevalent throughout the region [15]. Recently, the presence of ChLCB, a non-malvaceous betasatellite along with CLCuMB, a malvaceous betasatellite has been reported from two wild species of cotton [16]. We have also reported the association of ChLCB with a cotton leaf curl disease complex in which an African cotton begomovirus, *Cotton leaf curl Gezira virus *(CLCuGV) along with CLCuMB was found in commercially grown cotton in the province Sindh, Pakistan [17]. Recently, we also found ChLCB in some isolates of CLCuD in Punjab with very severe disease phenotype (unpublished data).

### The study

The following study was initiated to explore the contribution of βC1 gene encoded by ChLCB in the induction of cotton leaf curl disease symptoms. βC1 gene of ChLCB was expressed from a PVX vector. The DNA sequence coding for ChLCB was PCR amplified from ChLCB clone NGVB (accession number FR751147) using specific primers [17]. The PCR product of the expected size (approximately 450 bp) was cloned in to the plasmid vector pTZ57R/T using InsT/A cloning kit (Fermentas, USA). The cloned product was restricted with *Sal*I and *Cla*I and was subcloned in the sense orientation in PVX vector pgR107 [18]. The resulting construct (PVX^C.βC1^) was transformed into *Agrobacterium tumefaciens *strain GV3101 by electroporation [19]. *Nicotiana benthamiana *plants were agro-inoculated with the PVX^C.βC1 ^alone and or with CLCuGV and CLCuMV.

**Figure 1:**
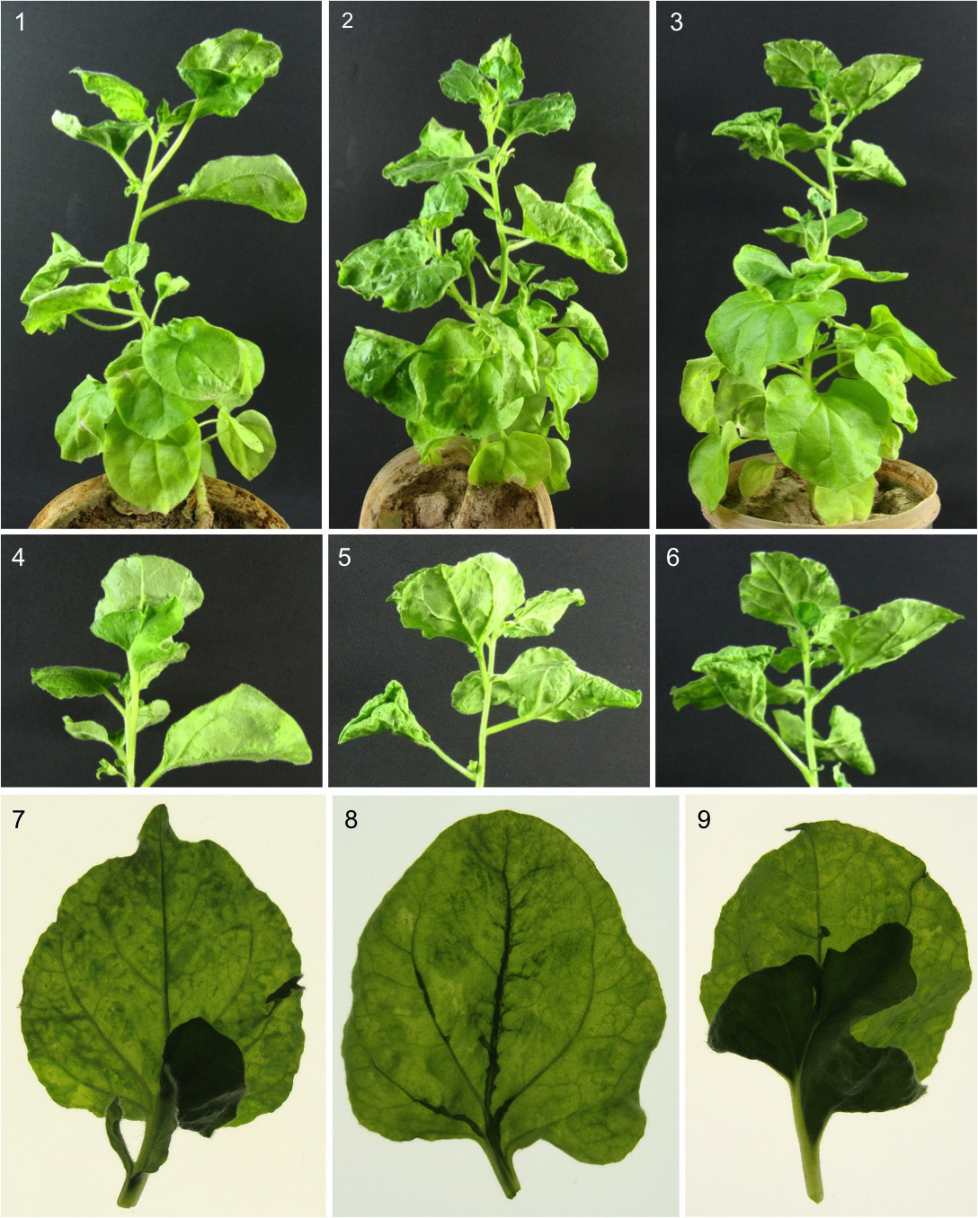
**Leaf curling and leaf-like enations developed by the expression of βC1 of ChLCB**. Panels 1-3 showing symptoms of PVX^C.βC1 ^in *Nicotiana benthamiana *plants and panels 4-6 showing the close up views of the same plants. Panel 7-9 show the close up views of underside of leaves using transmitted light. The close up shows the typical leaf enation developed by expression of βC1 of ChLCB.

The *N. benthamiana *plants inoculated with pgR107 showed typical symptoms of systemic PVX infection (data not shown) as reported earlier [20]. However, inoculation of *N. benthamiana *with PVX^C.βC1 ^resulted in typical disease symptoms of cotton leaf curl disease such as leaf curling and enations (Figure 1). Initially after 10-14 days all the inoculated plants (8/8) showed mild leaf curling in systemic leaves. After 22-25 days, plants developed typical symptoms induced by βC1, consisting of leaf curling, vein swelling and enations on the underside of the leaves, which frequently developed into leaf-like structure, mainly along midrib (Figure 1). These symptoms are identical to the symptoms induced by βC1 of CLCuMB when expressed from PVX vector [21]. These results indicate that the expression of βC1 of ChLCB from PVX vector resulted in development of full-range of typical symptoms of CLCuD in the absence of helper virus and without the backbone of betasatellite. The co-infiltration of PVX βC1 with CLCuGV and CLCuMV or prior infections with CLCuGV and CLCuMV followed by PVX βC1 (8 plants in each case) also resulted in the development of CLCuD symptoms including enations in *N. benthamiana *(data not shown).

The data presented here shows that the expression of βC1 of ChLCB from PVX vector develops a phenotype which qualitatively resembles phenotype induced by CLCuMB. Interestingly, the phenotype induced by the expression of Tomato yellow leaf curl China betasatellite also resulted in development of very similar phenotype [22]. The inoculation of CLCuGV with Cotton leaf curl Gezira betasatellite also developed a phenotype similar to that induced by cotton leaf curl disease complex prevalent in the Indian subcontinent [23]. A comparison of nucleotide sequences of these βC1 share low DNA sequence identity (19% only). It will be therefore interesting to compare protein domains that may be conserved and are important for phenotype produced by these apparently diverse βC1 proteins.

## Competing interests

The authors declare that they have no competing interests.

## Authors' contributions

MNT performed the experiments. SM conceived the study. MNT and SM wrote the manuscript. Both authors read and approved the final manuscript.
